# Empathy Modulates the Evaluation Processing of Altruistic Outcomes

**DOI:** 10.3389/fpsyg.2018.00407

**Published:** 2018-04-04

**Authors:** Xin Liu, Xinmu Hu, Kan Shi, Xiaoqin Mai

**Affiliations:** Department of Psychology, Renmin University of China, Beijing, China

**Keywords:** empathy, outcome evaluation, event-related potential (ERP), feedback-related negativity (FRN), P3, altruism

## Abstract

Empathy plays a central role in social decisions involving psychological conflict, such as whether to help another person at the cost of one’s own interests. Using the event-related potential (ERP) technique, the current study explored the neural mechanisms underlying the empathic effect on the evaluation processing of outcomes in conflict-of-interest situations, in which the gain of others resulted in the performer’s loss. In the high-empathy condition, the beneficiaries were underprivileged students who were living in distress (stranger in need). In the low-empathy condition, the beneficiaries were general students without miserable information (stranger not in need). ERP results showed that the FRN was more negative-going for self no-gain than self gain, but showed reversed pattern for other’s outcome (i.e., more negative for gain than no-gain) in the low-empathy condition, indicating that participants interpreted the gain of others as the loss of themselves. However, the reversed FRN pattern was not observed in the high-empathy condition, suggesting that the neural responses to one’s own loss are buffered by empathy. In addition, the P3 valence effect was observed only in the self condition, but not in the two stranger conditions, indicating that the P3 is more sensitive to self-relevant information. Moreover, the results of subjective rating showed that more empathic concern and altruistic motivation were elicited in the high-empathy condition than in the low-empathy condition, and these scores had negative linear correlations only with the FRN, but not with the P3. These findings suggest that when outcomes following altruistic decisions involve conflict of interest, the early stage of the processing of outcome evaluation could be modulated by the empathic level.

## Introduction

In our daily life, humans are sometimes required to make difficult social decisions involving benefit conflict between themselves and other social agents, such as whether they are willing to sacrifice personal benefit on behalf of a stranger’s welfare (Rilling and Sanfey, 2011). Numerous studies have focused on the inner mechanisms underlying the processing of such altruistic decisions which defined as increasing the welfare of others at a cost of the self (Batson and Shaw, 1991; de Waal, 2008), and found that multiple motivational and emotional factors, such as kin selection (Hamilton, 1964), reciprocal relation (Trivers, 1971), and empathic concern (Batson, 2008), could give rise to prosocial decisions. However, little is known about how people evaluate the consequent outcomes after they made altruistic decisions. Given that humans use positive or negative feedback to guide their next behaviors (Nieuwenhuis et al., 2004; Yang et al., 2015), it is necessary to understand the neural mechanisms underlying the processing of evaluating altruistic outcomes when self-interests are sacrificed.

Previous studies using the event-related potential (ERP) have found two ERP components related to the processing of outcome evaluation: the feedback-related negativity (FRN) and P3 (Schupp et al., 2000; Gehring and Willoughby, 2002; Miltner et al., 2014). The FRN, sometimes also called medial frontal negativity (MFN), originates from the medial-frontal cerebral regions (Holroyd and Coles, 2002; Nieuwenhuis et al., 2004; Wu et al., 2017), especially the anterior cingulate cortex (ACC) a brain area playing a central role in empathic responses for other person’s pain (Bernhardt and Singer, 2012). Accumulating studies have found that the FRN is more negative for the unfavorable outcomes than for the favorable outcomes, and reaches maximum between 200 and 300 ms following the onset of feedback stimuli (Gehring and Willoughby, 2002; Hajcak et al., 2005; Hauser et al., 2014; Paul and Pourtois, 2017). Furthermore, an enhanced FRN indicates the result being worse than expected (Holroyd and Coles, 2002; Nieuwenhuis et al., 2004) and reflects stronger motivational impact of the current stimuli (Masaki et al., 2006; Itagaki and Katayama, 2008; Luo et al., 2015). The P3 is a positive, large-amplitude potential with typical peak in the period of 300–600 ms after the onset of stimuli. It is larger for the positive feedback than for the negative feedback and for a large reward than for a small reward (Holroyd et al., 2006; Hewig et al., 2011; Peterburs et al., 2017). The P3 is generally believed to be related to the allocation of cognitive resources and the processing of attentional distribution (Polich, 1987, 2007; Yang et al., 2015; Hu et al., 2017), especially self-relevant attentional allocation (Gray et al., 2004; Linden, 2005). Extensive research regarding outcome evaluation suggests that the two ERP components could represent not only the evaluating processes of self-related outcomes but also those of other-related outcomes (e.g., Kang et al., 2010; Leng and Zhou, 2010; Ma et al., 2011; Wang et al., 2014; Hu et al., 2017). When the outcomes of other people have nothing to do with participants’ own benefit, the similar neural responses were observed in both self and other outcome conditions (Yu and Zhou, 2006; Leng and Zhou, 2014; Zhu et al., 2016). For example, in a pioneering work, Yu and Zhou (2006) asked participants to earn money in a gambling task for themselves and observe the reward/punishment feedback of others in which other’s outcomes were irrelevant to participants’ own interests. The results showed that the FRN was more negative-going to the loss outcome whenever outcomes related to self or to others, indicating that the FRN effect was elicited not only in self-evaluation condition, but also in other-evaluation condition. In other words, when there was no conflict of interests between oneself and others, comparable neural activities of outcome evaluation were observed in both self and others’ losing situations.

However, when there are benefit conflict between performers and beneficiaries, the ERPs of outcome evaluation change in a reverse way (Fukushima and Hiraki, 2006; Itagaki and Katayama, 2008; Marco-Pallares et al., 2010). Marco-Pallares et al. (2010) compared the ERP responses to outcomes of gambling in different situations across three groups. In the neutral group, individuals simply observed the performer’s action and their own benefit was not affected by others. In the parallel group, observers gained or lost the same amount of money as the performer. Finally, in the reverse group, competing motivation was aroused because the gain of others led to a loss of the observer and vice versa. The results showed that the ERPs of evaluators in the reverse situation showed an inverse pattern compared to the neutral and parallel conditions, indicating that the neural responses of evaluators translated the gain of others into the loss for themselves. However, an interesting study by Fukushima and Hiraki (2006) suggested that the inversed neural responses in competing situation are probably modulated by the empathetic processes. In their study, participants were required to perform a gambling task with their friends in which the friends’ loss resulted in the gain of themselves. The results showed that the inversed FRN effect for loss trials was only elicited for participants with less empathic tendency, whereas the neural discrepancy between gain and loss vanished in individuals with more empathic trait. The author proposed that the individual difference in the FRN is probably based on the allocation between empathetic and utilitarian processing. It is further confirmed by some studies suggesting that there are individual differences in the capacity for empathy and which links to the differences in the brain structure (Rueckert and Naybar, 2008; Banissy et al., 2012; Christov-Moore et al., 2014).

In addition, evidence from behavioral studies has indicated that the decision-making in competing situation (i.e., interest conflict with other social agents) can be influenced by the level of empathy (Batson and Moran, 1999; Batson and Ahmad, 2001). In their studies, they manipulated the individual’s empathic emotions in the prisoner’s dilemma (PD) task to induce the high altruistic motivation and found that participants increased their prosocial behaviors to cooperate with others, even though the best strategy was defecting the other partner to guarantee the maximized personal gain. Taking these studies together, we can conclude that empathy has great impact on altruistic decision-making and may play an important role in evaluating processes.

The present study aimed to examine whether the level of empathy could modulate the ERP responses to outcome evaluation when there was conflict between self-interest and other-interest. We revised the classical gambling task (Gehring and Willoughby, 2002) and required each participant to perform it in three conditions: gambling for themselves (self condition) and for two strangers. One of the strangers was described as an underprivileged student living in distress (stranger-in-need condition), while the other one was depicted as a general student who was studying in a regular urban school (stranger-not-in-need condition). Based on the empathy-altruism hypothesis that people would feel strong empathy for others in need and in distress (Batson and Moran, 1999; Batson and Ahmad, 2001), we considered the stranger-in-need scenario as the high-empathy condition while the stranger-not-in-need scenario as the low-empathy condition. One point should be noted that psychological conflict was settled in two strangers’ situations in which participants had to pay the same amount of money from their remuneration as the amount they gained for others. Our hypotheses were that the FRN effect would inverse in the low-empathy condition, but would not inverse in the high-empathy condition. Further, the P3 effect would be observed only in the self condition given that P3 is more sensitive to self-related stimuli (Gray et al., 2004; Linden, 2005).

## Materials and Methods

### Participants

Thirty undergraduate and graduate students (15 females; mean age 21.27 ± 2.1 years) at Renmin University of China were recruited in the present study. All participants were right handed, had normal or corrected to normal vision, and reported no history of neurological or psychiatric diagnoses. The data of two male participants were excluded because there were not enough trials (less than 30 trials) after artifacts were removed (Marco-Pallares et al., 2011). Written informed consent was obtained from all participants. The study was approved by the Institutional Review Board of Department of Psychology at Renmin University of China.

### Procedure

At the beginning of the experiment, each participant was instructed to play the gambling game three times for different beneficiaries, including himself/herself and two strangers. In the high-empathy condition, the beneficiary would be an underprivileged student who came from a school in remote poverty regions (stranger in need). In the low-empathy condition, the reward receiver would be a general student who was studying in a normal urban school (stranger not in need). All the participants were informed that they would get the amount of money they gained when they played for themselves. However, when the participant played games for two strangers, the beneficiaries would receive the money they won in the game as the prize, and the participant would lose the same amount of money. All participants were informed how much money they earned for themselves and strangers after the experiment was over. Ultimately, they were paid an amount of money between 60 and 65 Chinese yuan.

**FIGURE 1:**
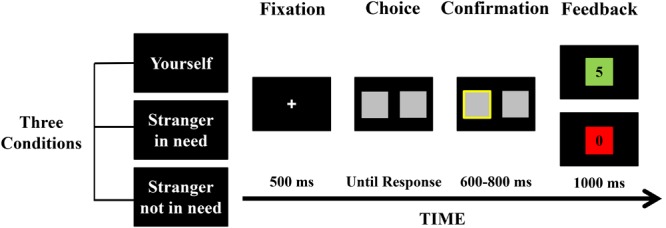
An illustration of a single trial in the gambling task. Each trail began with a fixation cross. Participants viewed two gray cards without numeral cue and were required to choose one of them by pressing the corresponding key. Their choice was then highlighted for 600–800 ms. After that, the outcome feedback was presented for 1000 ms.

Participants were seated comfortably in front of a computer screen in an electrically isolated room. They were asked to play the gambling game adapted from the task designed by Gehring and Willoughby (2002). As illustrated in **Figure 1**, each trial began with a white fixation cross presented for 500 ms on a black background. Then, two gray cards were presented on either side of the fixation point with no numeral cue on them. Participants were required to choose between the two alternatives by pressing a corresponding response button (F or J key on the keyboard) with their left or right index finger. When the participant responded, the chosen card was highlighted by a thickening of a yellow border for 600–800 ms, and then the outcome (5 or 0) behind the chosen card shown centrally was displayed for 1000 ms. The inter-trail interval was 600–800 ms. To increase the salience of the valence of the outcome, the chosen card turned red/green color to indicate gain/no-gain outcomes and the colors of cards were counterbalanced among participants. In the situation that participants played the game for themselves, the numeral 5 means that participants gained 5 points and 0 means that participants gained no points. In the situation that participants played the game for strangers, 5 means that strangers gained 5 points but participants themselves lost 5 points; 0 means that strangers gained no points and participants did not lost points either. According to previous research, the FRN is determined by the value of the outcome relative to the range of other possible outcomes in the task, rather than by the objective value of the outcome (Holroyd et al., 2004). We thus expected that no-gain feedback could elicit the FRN effect as same as loss feedback did.

There were 270 trials in total, divided into three blocks with 90 trails and only one of three beneficiary conditions in each block. At the beginning of each block, participants were informed that which beneficiary they would play for in this block, and were emphasized to notice the meaning of winning money in this block. Unknown to the participants, the gain/no-gain feedback was manipulated according to a random sequence, and each participant received equal times of each feedback condition. The order of the three blocks was counterbalanced over participants.

The stimuli were presented by E-prime 2.0 software package (PST, Pittsburgh, PA, United States). The formal experiment started after 5 trials of practice for each participant. After finishing the gambling task, the participants firstly filled out the Chinese version of the Self-Report Altruism Scale (C-SRA scale) (Rushton et al., 1981; Chou, 1996), a paper questionnaire that contains 20 statements to measure altruism in a behaviorally concrete manner. Then, they were asked to complete a 5-point scale to rate their subjective “motivation” to win the game and “empathic feeling” about the outcome. Specifically, they were asked to rate how much they were willing to play the game (1 = “not at all” to 5 = “very much”), how much they were willing to win in the game (1 = “not at all” to 5 = “very much”), and what they felt about the winning outcomes (1 = “very unhappy” to 5 = “very happy”) for themselves, the stranger in need, and the stranger not in need, respectively. The first question measured the general “motivation” of participants to make efforts on this task and the second one measured the specific “motivation” to increase welfare of self and others. The scores of the former two questions were clumped together to create a composite measure for the “motivation” to win for each beneficiary. The last question measured whether participants felt positive or negative emotions when gaining money for themselves and for strangers, regarding as “empathic emotion” to others.

### EEG Recording and Analysis

EEG was recorded with NeuroScan synamp2 amplifier (Neuroscan Inc., Sterling, VA, United States), using an elastic cap with 64 tin electrodes according to the international 10/20 system. The signals were amplified with a band-pass filter of 0.01–100 Hz and continuously sampled at 1000 Hz/channel for the offline analysis. All rows of electrode recordings were referenced to an electrode placed over the left mastoid, and were re-referenced offline to the average of the left and right mastoids. The vertical and horizontal electrooculograms (EOGs) were collected with electrodes placed on the left supraorbital and infraorbital, and on the outer canthi of the left and right eyes respectively. All the interelectrode impedances were less than 5 kΩ.

The EEG data were processed offline using the Neuroscan 4.5 software. Ocular artifacts were corrected using a regression procedure implemented in the Neuroscan software (Semlitsch et al., 1986). Raw EEG data were segmented into epochs from 200 ms before to 800 ms after the onset of outcome feedback. The 200 ms preceding the feedback stimulus served as baseline. Epochs containing artifacts exceeding ± 75 μV were rejected from the analysis. The data were digitally low-pass filtered below 30 Hz and were then averaged for each condition.

The present analyses focused on the FRN and P3 elicited by outcome feedback. The FRN was measured as the mean amplitudes in the time window of 210–300 ms following the feedback presentation. The P3 was defined as the most positive peak in the window of 330–430 ms after the onset of feedback stimuli. Based on the topographical distribution of each ERP component and previous research (e.g., Yeung and Sanfey, 2004; Leng and Zhou, 2014), the FRN was preliminary calculated across 3 electrodes (Fz, FPz and Cz) and the P3 was quantified across 2 electrodes (CPz and Pz). The results indicated that the effect of FRN was greatest at the FCz site, and the effect of P3 was largest at the CPz site. Hence, we focused on the FCz and CPz electrodes for more detailed analyses at which the ERP effects were maximal.

The FRN and P3 data were each subjected to repeated measures analysis of variance (ANOVA) with two within-subject’s factors: Beneficiary (self vs. stranger-in-need vs. stranger-not-in-need) and Reward Valence (gain vs. no-gain). The significance level was set at 0.05 for all the statistical analyses. Bonferroni-corrected method was performed for *post hoc* testing of significant main effects, while simple effect analysis was using for testing significant interactions. Greenhouse–Geisser correction of the ANOVA assumption of sphericity was applied where appropriate. Effect size in all ANOVA analyses were reported by partial eta-squared ($\eta_{p}^{2}$), where 0.05 represents a small effect, 0.10 represents a medium effect, and 0.20 represents a large effect (Cohen, 1973). All the statistical analyses were performed by SPSS (23.0; SPSS, Inc., Chicago, IL, United States).

## Results

### Behavioral Results

A few trails with reaction time (RT) greater than 2000 ms were deleted as extreme value. In the gambling task, the mean (±SD) RTs for choice responses in three conditions were 431 ± 113 ms (self), 467 ± 110 ms (stranger-in-need), and 456 ± 137 ms (stranger-not-in-need), respectively. One-way ANOVA was used to compare the RTs among three beneficiaries. No significant difference was found among them [*F*(2,81) = 0.341, *p* = 0.7].

### Subjective Ratings

**FIGURE 2:**
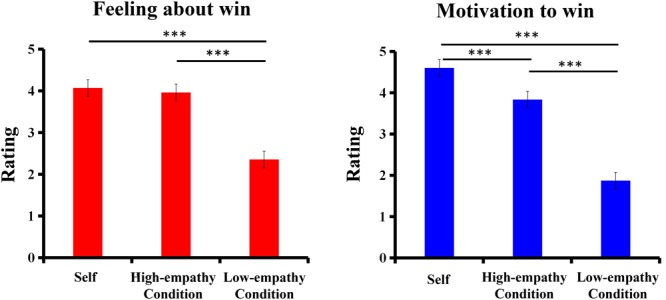
Subjective ratings for motivation to win and feelings about win. Error bars indicate SEM (standard error of the mean). ^∗∗∗^*p* < 0.001.

**Figure 2** shows the subjective ratings of feelings about win and motivation to win for each beneficiary. One-way ANOVA on the subjective rating of the feeling of empathy toward winning money for different beneficiaries (self vs. stranger-in-need vs. stranger-not-in-need) was conducted. The results revealed a significant effect of beneficiary, [*F*(2,81) = 44.26, *p* < 0.001, $\eta_{p}^{2}$ = 0.84]. Bonferroni-corrected *post hoc* test showed that participants felt happier when they getting reward for underprivileged students than general students (*p* < 0.001), while a similar positive feeling was found toward gaining money for themselves and for underprivileged students (*p* = 0.62). It indicated that participants experienced more empathic emotion in the high-empathy condition rather than in the low-empathy condition. One-way ANOVA on the subjective rating of motivation to win for the beneficiary (self vs. stranger-in-need vs. stranger-not-in-need) revealed a significant effect of beneficiary, [*F*(2,81) = 132.37, *p* < 0.001, $\eta_{p}^{2}$ = 0.93]. Bonferroni-corrected *post hoc* test showed that the motivation to win for the self (4.61) was higher than that for two strangers, (*ps* < 0.001), whereas the motivation to win for the stranger-in-need (3.84) was higher than that for stranger-not-in-need (1.87), (*p* < 0.001).

### The FRN Results

**FIGURE 3:**
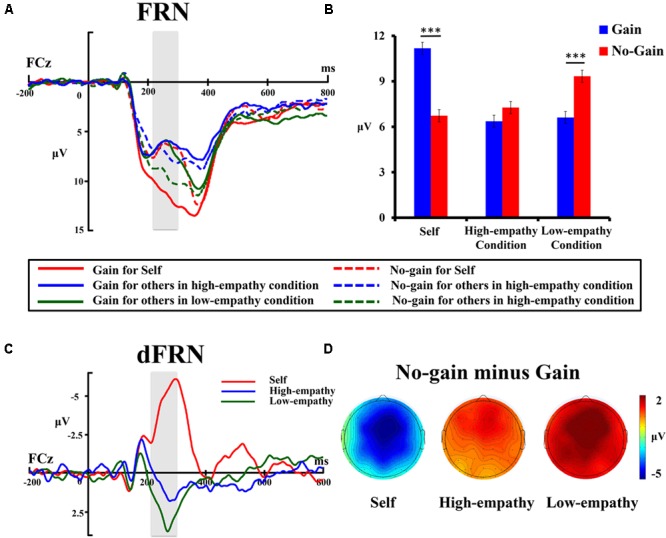
**(A)** Grand-average ERP waveforms from the FCz electrode site. The gray areas highlight the time window of the FRN (210–300 ms) used for statistical analysis. **(B)** The bar graphs show the mean value of the FRN amplitude for each condition. Error bars indicate standard error of the mean (SEM). ^∗∗∗^*p* < 0.001. **(C)** Difference waveforms of no-gain minus gain. The gray areas highlight the time window of the dFRN (210–300 ms) used for statistical analysis. **(D)**Topographic maps of different waveforms (no-gain minus gain) in the 210–300 ms time window for self, high-empathy, and low-empathy conditions.

**Figure 3A** shows grand-average ERP waveforms at the FCz site. The mean amplitude of FRN was analyzed by a 3 (Beneficiary: self vs. stranger-in-need vs. Stranger-not-in-need) × 2 (Reward Valence: gain vs. no-gain) repeated measures ANOVA. The results showed that the main effect of the beneficiary was significant [*F*(2,26) = 6.52, *p* < 0.01, $\eta_{p}^{2}$ = 0.33], indicating that the size of the FRN effect was different among the three beneficiary conditions. The main effect of reward valence was not significant [*F*(1,27) = 0.43, *p* = 0.5]. Moreover, the ANOVA revealed a significant interaction between Beneficiary and Valence [*F*(2,26) = 23.14, *p* < 0.001, $\eta_{p}^{2}$ = 0.64].

Further simple effect analyses were conducted to investigate the interaction. As we can see in **Figure 3B**, no-gain trials showed greater negativity than gain trials only for self condition (*p* < 0.001), while the typical pattern was reversed in trails for the outcomes of strangers. In the stranger-in-need condition, the FRN differentiation between gain and no-gain was remarkably diminished and no significant FRN difference was found between gain and no-gain (*p* = 0.31). On the other hand, in the stranger-not-in-need condition, the FRN difference between gain and no-gain outcomes was reversed, with more negative-going FRN for gain than no-gain outcomes (*p* < 0.001), indicating that participants regarded the gain for others as negative outcome (i.e., loss) for themselves only in the low-empathy condition.

In addition, we measured the mean amplitude of the FRN on the difference waves of no-gain minus gain (dFRN) for further repeated measures ANOVA. The dFRN for the participant’s personal performance (self-dFRN) was calculated as self-no-gain minus self-gain, while the dFRN for the strangers (other-dFRN) was calculated as the other’s no-gain minus the other’s gain. As **Figure 3C** showed, a significant main effect of beneficiary was found [*F*(2,26) = 23.14, *p* < 0.001, $\eta_{p}^{2}$ = 0.640]. Bonferroni-corrected *post hoc* test showed that the self-dFRN (-4.47 μV) was significantly more negative than two other-dFRNs (*p*s < 0.001), whereas the other-dFRN in the high-empathy condition (0.91 μV) was smaller than that in the low-empathy condition (2.73 μV), though only marginally significant (*p* = 0.06). Scalp topographies of the dFRN also revealed these differences among three conditions (**Figure 3D**).

Pearson correlation analysis was conducted between the FRN amplitudes and subjective assessment scores. The results showed that the FRN was negatively correlated with subjective scores of motivation (*r* = -0.282; *p* < 0.001) and empathic emotion (*r* = -0.336; *p* < 0.001), indicating that the more the participants motivated to win or felt affect to the other’s outcomes, the more the FRN enhanced. However, no correlation was found between the FRN amplitude and self-report altruism scale (*p* = 0.518).

### The P3 Results

**FIGURE 4:**
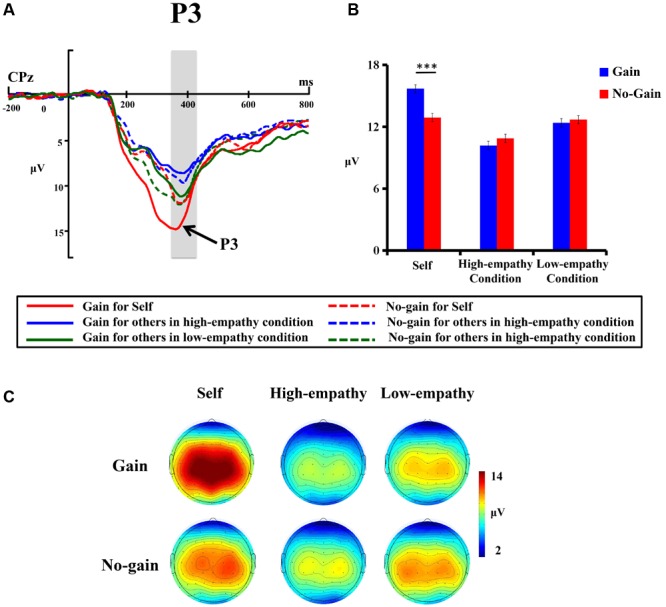
**(A)** Grand-average ERP waveforms from the CPz electrode site. The gray areas highlight the time window of the P3 (330–430 ms) in which the peak amplitude was measured. **(B)** The bar graphs show the mean value of the P3 amplitude for each condition. Error bars indicate standard error of the mean (SEM). ^∗∗∗^*p* < 0.001. **(C)** Topographic maps of the P3 for the self, high-empathy, and low-empathy conditions.

**Figure 4A** shows grand-average ERP waveforms at CPz electrode site. The peak amplitude of P3 at CPz was analyzed by a 3 (Beneficiary: self vs. stranger-in-need vs. Stranger-not-in-need) × 2 (Reward Valence: gain vs. no-gain) repeated measure ANOVA. The main effect of beneficiary was significant [*F*(2,26) = 15.19, *p* < 0.001, $\eta_{p}^{2}$ = 0.54], but the effect of reward valence was not found [*F*(1,27) = 2.98, *p* = 0.09]. Bonferroni-corrected *post hoc* test showed that the P3 was larger in the self condition than in both stranger conditions (*p*s < 0.001), while the P3 amplitude was the smallest in the high empathy condition (*p*s < 0.05). More importantly, the ANOVA revealed a significant interaction between Beneficiary and Valence [*F*(2,26) = 7.29, *p* < 0.001, $\eta_{p}^{2}$ = 0.36]. Further simple effect analysis was conducted to examine this interaction. As we can see in **Figure 4B**, the results revealed that the gain feedback induced a larger P3 than the no-gain did only in the self condition (*p* < 0.001), but this P3 difference between gain and no-gain feedback was not observed in the other two conditions. Scalp topographies of the P3 also revealed these differences among three conditions (**Figure 4C**).

Pearson correlation analysis was also conducted between the P3 amplitudes and subjective assessment scores. However, no significant correlation was found between P3 with either subjective scores of motivation (*p* = 0.11), empathic emotion (*p* = 0.15), or the rating of self-report altruism (*p* = 0.29).

## Discussion

In this study, using the gambling task in which participants made money for themselves and two strangers, we examined the neural correlates of empathy modulating the evaluation of outcomes that involved benefit conflict. The ERP results showed that an inversed FRN effect occurred when evaluating another person’s outcomes in the low-empathy condition, but did not appear in the high-empathy condition. Further, the P3 was larger for the gain outcome than the no-gain outcome in the self condition, but did not show the valence effect in the two stranger conditions. The results of the present study suggest that empathy could modulate the neural responses to altruistic outcomes in which increasing welfare of others could result in a cost of the self.

The FRN was more negative-going to no-gain than to gain when gambling for self, but reversed in opposite polarity when gambling for others in the low-empathy condition. This finding is consistent with previous studies in which they found a negative-going FRN for antagonist’s gain, as if gains of others were interpreted as losses of oneself (Fukushima and Hiraki, 2006; Itagaki and Katayama, 2008; Marco-Pallares et al., 2010). Given that the FRN elicited by self-outcome (self-FRN) represented the motivational/affectional impact of the outcomes (Gehring and Willoughby, 2002), our results provide a direct evidence to the theory that the FRN elicited by other’s outcome (other-FRN) also reflects the response of inner meanings of positive/negative stimuli. Previous studies have reported that when other’s outcomes did not relate to one’s own benefit, the other-FRN showed the same polarity as the self-FRN (Yu and Zhou, 2006; Fukushima and Hiraki, 2009; Kang et al., 2010; Ma et al., 2011; Leng and Zhou, 2014), indicating that the neural activities of evaluating other’s outcomes are comparable with those of evaluating one’s own. However, the circumstances become complicated when the interests of self conflict with that of others. Based on the ideally defined hypothesis in traditional economics that people are generally maximizing their own interests, it was not surprising that the FRN was more positive-going to no-gain than to gain when gambling for strangers in low empathy condition, indicating that individuals evaluated the outcomes of decisions depending on their own motivation, and regarded the gain of others as the loss of self in the interest-competing context.

Critically, as we expected, the other-FRN was not reversed in the high-empathy condition, and showed no difference between other’s gain and no-gain. It might suggest that the neural activities in the low-empathy condition are sensitively elicited by other’s gains, while the neural responses to other’s outcomes are inhibited in the high-empathy condition. We believe that the different patterns of FRN between the low- and high-empathy condition may be attributed to the buffer function of empathy. Behavioral studies have found that empathy, an ability to infer and share the mental and emotional states of others (Preston and de Waal, 2002; Lamm et al., 2011), can induce altruistic motivation to increase other’s welfare and improve more prosocial behaviors (Eisenberg and Fabes, 1990; Batson, 2008). Subsequently, the findings in neuro-imaging studies provided a neural substrate perspective to understand the effect of empathy on altruistic decisions. Using the functional magnetic resonance imaging (fMRI), a number of studies have found that perceiving others’ affective states would activate neural network involving in the first-hand experience of these states called “shared representative network” (Singer et al., 2004; Cacioppo and Decety, 2009; Lamm et al., 2011; Marsh et al., 2014). For example, Singer et al. (2004) asked volunteers to observe their lovers who could elicit their highest level of empathy for suffering pain. The results showed that the brain areas, such as the anterior insula (AI) and dorsal-anterior midcingulate cortex (dACC), were activated in both direct pain and vicarious pain situations. Later, Mobbs et al. (2009) extended pain empathy to social emotions by contrasting the neural responses to the socially desirable others getting reward vs. to directly gaining money for themselves. They found a similar reward mechanism employed in both situations, confirming that the “shared representation network” could apply to complex social emotions elicited by favorable or unfavorable outcomes. Taking these findings together, the corresponding neural network would be evoked in individuals who are induced high empathy, which makes them be more likely to experience the other’s feeling. Therefore, in the high-empathy condition of the present study, individuals would feel internal pain for needy students and have a strong altruistic motivation to help them, which could counteract the suffering of their own loss. We thus observed, a decreased FRN when participants evaluated other’s gain that led to the loss of themselves.

The finding of P3 showed a main effect of beneficiary in which the P3 amplitudes were larger in the self condition than in the two stranger’s conditions. This is consistent with previous finding (Ma et al., 2011) that the mean amplitude of P3 was larger for the self-execution than for friends or strangers. Interestingly, the finding that P3 was larger in the low empathy condition than in the high empathy condition did not congruent with the recent works of Leng and Zhou (2010, 2014) who found that P3 was more positive for the friends than for the strangers. Since the P3 reflects the allocation of cognitive resources (Polich, 1987, 2007; Yang et al., 2015; Hu et al., 2017), these results suggest that the larger P3 indicates that more resources are allocated to the ongoing task. As we expected, the most cognitive resources were used to evaluate self-related feedback in order to maximize one’s own profits. However, when the interests were conflict between oneself and others, cognitive load was increased to balance two competing motivations, egoistic motives and altruistic motives. In other words, the cognitive resources of outcome evaluation were affected by the processing of empathy. Therefore, the P3 was smallest in high empathy condition than in low empathy condition indicating that more cognitive resources were occupied by processes of empathic concern and conflict management.

In addition, the valence effect of P3 was only observed in the self-condition, but disappeared in both high-empathy and low-empathy conditions. Such inapparent valence effect on other’s feedback was consistent with the findings of previous studies on neural processes of outcome evaluation when the interest of oneself was conflict with that of others (Fukushima and Hiraki, 2006; Itagaki and Katayama, 2008; Leng and Zhou, 2014). Moreover, the P3 amplitudes did not covary with subjective scores of empathy nor motivation, suggesting that different from the FRN, the P3 effect of outcomes was not modulated by empathy nor motivation. Given that the P3 effect was only observed in self-related feedback rather than in other-related feedback, we thus suggest that the P3 reflects an allocation of attentional resources that may distinguish between “self” and “others.” This interpretation can also be supported by the previous studies which found that P3 was larger for self-relevant stimuli relative to control stimuli (Gray et al., 2004; Tacikowski and Nowicka, 2010), suggesting that P3 is an index of the allocation of attentional resources, and evokes by autobiographical stimuli, instead of empathic emotion.

Moreover, we found that the subjective score of empathic emotion correlated with FRN, but did not covary with P3, indicating that empathy play a central role in the early stage of neural processes when we evaluating other’s outcomes. However, no significant correlation was found between the rating of self-report altruism with either the FRN or the P3, suggesting that the individual difference in altruistic trait have no effect on the processes of outcome evaluation. These results together may support the hypothesis that the neural mechanism underlying empathy could be independent of that underlying altruistic tendencies (Tankersley et al., 2007).

In sum, the current study investigated the neural mechanism of how empathy modulates outcome evaluation toward others in a gambling task involving conflict between self and other interest. A reversed FRN effect was elicited for strangers only in the low-empathy condition, whereas such FRN pattern was not observed in the high-empathy condition. These findings indicate that the neural processes for other’s outcomes are modulated by individuals’ empathy levels. Specifically, the high level of empathy could let people think from the perspective of others and induce a stronger altruistic motivation which counteracts with the egoistic motivation. These findings support previous studies showing that empathy could promote prosocial decision-making and cooperative behaviors (Batson and Ahmad, 2001; Smith, 2006; Christov-Moore et al., 2014) and provide the underlying neural evidence to help us understand prosocial behaviors better. In addition, there was the P3 valence effect only in the self condition, but not in the two stranger conditions, regardless of the levels of empathy, indicating that P3 is more sensitive to the distribution of attention resource in self-relevant information.

There are limitations in the present study. We manipulated the level of empathy through impoverishing strangers, which might result in the activation of an altruistic motivation. Thus it is hard to exclude the influence of motivation on the evaluation processing of other’s outcomes in the present study. In the future studies, it would be worthwhile to separate the two important factors: altruistic motivation and empathy, and differentiate their influences on the evaluation of other’s outcomes. In addition, accumulating evidence has shown that there are differences in the capacity of empathy between females and males (Schirmer et al., 2007; Gardner et al., 2012; Christov-Moore et al., 2014) and among individuals with different social value orientations (Declerck and Bogaert, 2008). Fukushima and Hiraki (2006) also found that the discernable MFN to the opponent’s outcomes only emerged for female participants, but not for males. Therefore, the individual difference of empathy modulating outcome evaluation is a very interesting issue, which is worth further research in the future. Moreover, the ecological validity of the current experimental design may need to be improved. In our daily life, people usually make decisions and evaluate outcomes in more complex social contexts. Other individual’s attitudes and behaviors also have impacts on how we evaluate other’s outcomes. These factors should also be considered in the future studies.

## Author Contributions

XL and XM designed the study. XL and XH collected and analyzed the data. XL wrote the manuscript. XM, KS, and XH edited the manuscript. All authors reviewed the manuscript.

## Conflict of Interest Statement

The authors declare that the research was conducted in the absence of any commercial or financial relationships that could be construed as a potential conflict of interest.
